# *Cis* and *trans* interactions between genes encoding PAF1 complex and ESCRT machinery components in yeast

**DOI:** 10.1007/s00294-018-0828-6

**Published:** 2018-03-22

**Authors:** Joana Rodrigues, David Lydall

**Affiliations:** Institute for Cell and Molecular Biosciences, Newcastle University Medical School, Newcastle upon Tyne, NE2 4HH UK

**Keywords:** *Saccharomyces cerevisiae*, PAF1 complex, ESCRT machinery, Adjacent gene effect, Telomeres

## Abstract

*Saccharomyces cerevisiae* is a commonly used model organism for understanding eukaryotic gene function. However, the close proximity between yeast genes can complicate the interpretation of yeast genetic data, particularly high-throughput data. In this study, we examined the interplay between genes encoding components of the PAF1 complex and *VPS36*, the gene located next to *CDC73* on chromosome XII. The PAF1 complex (Cdc73, Paf1, Ctr9, Leo1, and Rtf1, in yeast) affects RNA levels by affecting transcription, histone modifications, and post-transcriptional RNA processing. The human PAF1 complex is linked to cancer, and in yeast, it has been reported to play a role in telomere biology. Vps36, part of the ESCRT-II complex, is involved in sorting proteins for vacuolar/lysosomal degradation. We document a complex set of genetic interactions, which include an adjacent gene effect between *CDC73* and *VPS36* and synthetic sickness between *vps36Δ* and *cdc73Δ, paf1Δ*, or *ctr9Δ*. Importantly, *paf1Δ* and *ctr9Δ* are synthetically lethal with deletions of other components of the ESCRT-II (*SNF8* and *VPS25*), ESCRT-I (*STP22*), or ESCRT-III (*SNF7*) complexes. We found that RNA levels of *VPS36*, but not other ESCRT components, are positively regulated by all components of the PAF1 complex. Finally, we show that deletion of ESCRT components decreases the telomere length in the S288C yeast genetic background, but not in the W303 background. Together, our results outline complex interactions, in *cis* and in *trans*, between genes encoding PAF1 and ESCRT-II complex components that affect telomere function and cell viability in yeast.

**Electronic supplementary material:**

The online version of this article (10.1007/s00294-018-0828-6) contains supplementary material, which is available to authorized users.

## Introduction

The budding yeast *Saccharomyces cerevisiae* is the best understood eukaryotic model organism. Its genome is compact and composed of over 6000 genes in 12 Mb of DNA, approximately 100-fold less dense than the human genome (Herrero et al. 2016). Such a high density of genes in yeast often leads to adjacent gene effects where deletion of one gene affects the function of the adjacent gene, for example by interfering with 5′ UTR (untranslated regions) or 3′ UTRs sequences (Ben-Shitrit et al. 2012; Atias et al. 2016). Adjacent gene effects can confound interpretation of genetic data, especially in large-scale studies. In yeast, *CDC73*, encoding a component of the PAF1 complex, is adjacent to *VPS36*, encoding a component of the ESCRT machinery and the two genes have been suggested to show an adjacent gene effect (Ben-Shitrit et al. 2012).

The PAF1 complex is a conserved transcriptional elongation factor that binds RNA polymerase II and is comprised of Cdc73, Paf1, Ctr9, Leo1, and Rtf1 (Betz et al. 2002; Jaehning 2010; Tomson and Arndt 2013). In budding yeast, Cdc73 and Rtf1 are the main interfaces for the physical interaction between the PAF1 complex and RNA pol II (Nordick et al. 2008; Xu et al. 2017). The PAF1 complex affects transcript abundance (Squazzo et al. 2002; Chen et al. 2009; Kim et al. 2010; Crisucci and Arndt 2011; Xu et al. 2017), histone modifications (Krogan et al. 2003a), and poly(A) site utilization (Penheiter et al. 2005).

In yeast, there is evidence that the PAF1 complex affects telomere function. Specifically, *cdc73Δ, paf1Δ*, and *ctr9Δ* cells show reduced levels of TLC1 RNA (the RNA template of telomerase) (Mozdy et al. 2008). In addition, Paf1 and Ctr9, but not Cdc73, are required to maintain low levels of TERRA (telomere repeat containing RNA) (Rodrigues and Lydall 2018). Finally, genetic screens have shown that Cdc73, Leo1, and Rtf1 are important for the fitness of telomere defective cells (carrying a *cdc13-1* temperature-sensitive allele of the telomere capping gene *CDC13*) (Addinall et al. 2011).

It is interesting to note that although the five proteins of the PAF1 complex associate with RNA pol II, deletion of individual PAF1 complex components in yeast leads to distinct phenotypes (Betz et al. 2002; Rodrigues and Lydall 2018). Besides the already mentioned differences in TLC1 and TERRA levels in strains lacking different PAF1 complex components, there is also a strong difference in fitness amongst the different PAF1 deletion mutants. For example, *ctr9Δ* and *paf1Δ* cause severe growth defects, while *cdc73Δ* and *rtf1Δ* cause mild growth defects and *leo1Δ* causes no growth defects (Betz et al. 2002; Rodrigues and Lydall 2018). There are at least three plausible, not mutually exclusive, explanations for why deletion of different PAF1 complex components lead to distinct phenotypes: (1) the existence of subcomplexes; (2) complex components perform specific and independent functions; and/or (3) the existence of an adjacent gene effect.

Data from numerous experimental systems support the idea that the different PAF1 complex components perform different functions and the more severe defects of cells lacking Paf1 or Ctr9 have been attributed to the scaffolding properties of these components (Nordick et al. 2008; Tomson and Arndt 2013; Xu et al. 2017; Rodrigues and Lydall 2018). Indeed, structural studies in human and yeast cells have revealed Ctr9 to be the main scaffold protein for the PAF1 complex, and loss of Paf1 causes a significant decrease in Ctr9 protein levels (Nordick et al. 2008; Chu et al. 2013; Xu et al. 2017). However, it has also been suggested that a complete *CDC73* deletion, often used in yeast studies, is affecting the function of *VPS36*, one of its adjacent genes (Ben-Shitrit et al. 2012). Large-scale surveys have shown that *cdc73Δ* and *vps36Δ* strains share many characteristics, for example reduced telomere length (Askree et al. 2004; Rog et al. 2005; Dieckmann et al. 2016), heat sensitivity (Betz et al. 2002; Sinha et al. 2008), and decreased resistance to hygromycin B and hydroxyurea (Betz et al. 2002; Dudley et al. 2005; Fell et al. 2011; Ejzykowicz et al. 2017).

**Fig. 1:**
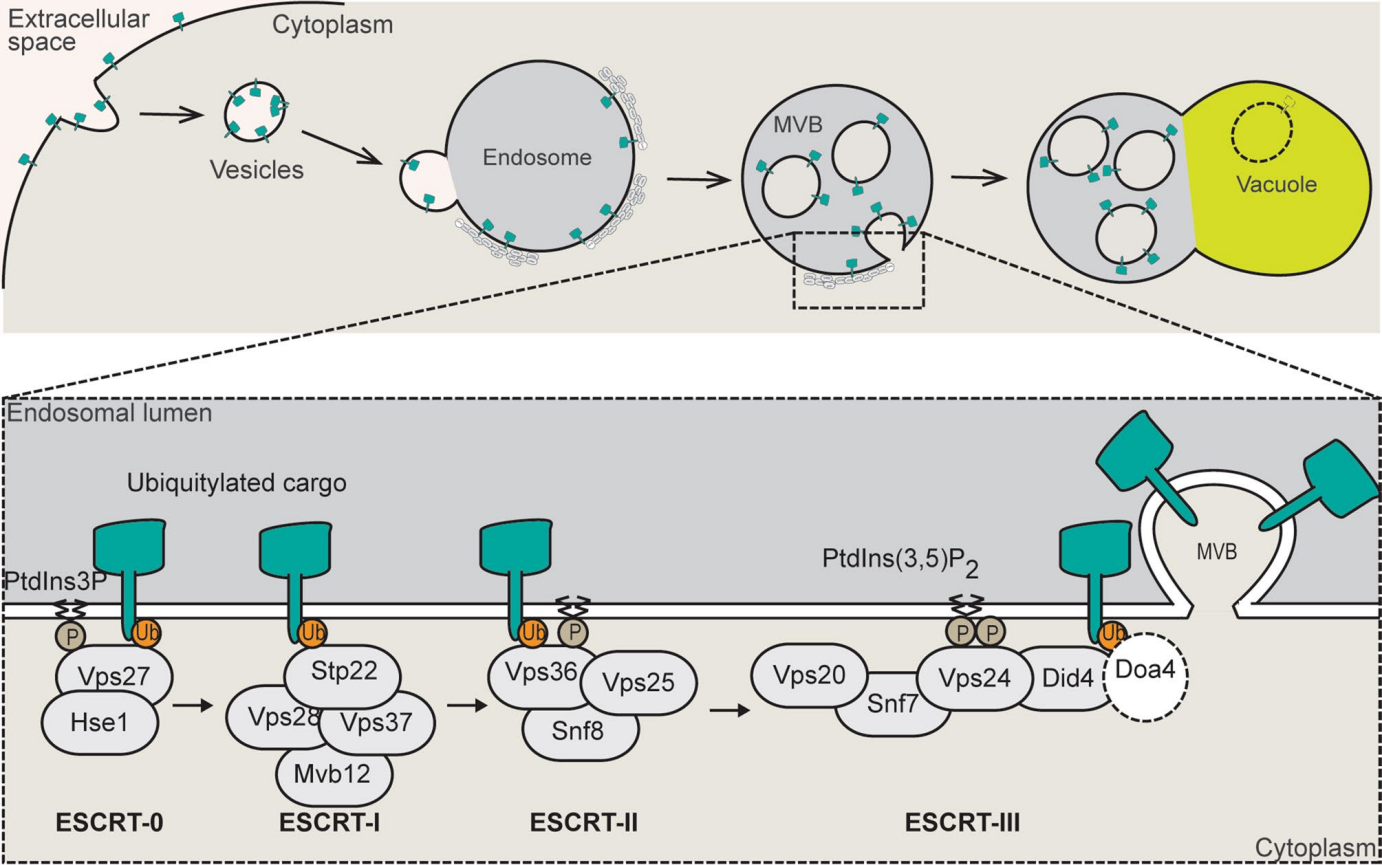
ESCRT machinery role in the multivesicular body pathway. Cartoon showing the function of the yeast ESCRT machinery in the sorting of ubiquitylated membrane proteins (cargo) for degradation through the multivesicular body (MVB) pathway. The ESCRT machinery is composed of ESCRT-0 (Vps27 and Hse1), ESCRT-I (Stp22, Mvb12, Vps37, and Vps28), ESCRT-II (Snf8, Vps25, and Vps36), and ESCRT-III (Snf7, Vps20, Vps24, and Did4). Doa4, an ubiquitin-specific protease, is not classified as part of the ESCRT machinery but is part of the MVB pathway. ESCRT-0 recognises Phosphatidyl Inositol 3 phosphate (PtdIns3P) and ubiquitylated cargo in the endosomal membrane. The remaining ESCRT complexes (-I, -II, and -III) sequentially interact with the cargo leading to the cargo deubiquitylation and formation of vesicles inside the endosome (MVB). The vesicles and their cargo are degraded when the MVB fuses with the vacuole/lysosome. Adapted from (Williams and Urbe 2007; López-Reyes et al. 2011; Schmidt and Teis 2012; Schuh and Audhya 2014)

*VPS36* is widely conserved among eukaryotes and encodes a protein that is part of the ESCRT (Endosomal Sorting Complexes Required for Transport)-II complex (Teo et al. 2004). The main function for the ESCRT machinery (ESCRT-0, ESCRT-I, ESCRT-II and ESCRT-III) is in the remodelling of membranes, which is important to its role in the multivesicular body (MVB) pathway of protein degradation (Schmidt and Teis 2012) (Fig. 1). The MVB pathway sorts ubiquitylated membrane proteins for degradation in vacuoles/lysosomes (Schmidt and Teis 2012). During this process, each of the ESCRT complexes assembles on endosomes in a sequential manner (Schmidt and Teis 2012). Together with MVB formation, the ESCRT machinery contributes to other membrane scission events like retroviral particle release and midbody abscission during cytokinesis (Caballe and Martin-Serrano 2011). Finally, the ESCRT machinery was also suggested to promote gene transcription due to its presence at the 3′ end of coding regions, and the increased sensitivity to 6-azauracil of *ESCRT* mutants (Song et al. 2014).

In this study, we demonstrate an adjacent gene effect between *CDC73* and *VPS36*, and its effect in the fitness of telomere defective cells. We describe a number of complex interactions between the components of the PAF1 complex and *VPS36* in budding yeast. We report, for the first time, synthetic lethal genetic interactions between *VPS36* and two components of the PAF1 complex: *PAF1* and *CTR9*. Importantly, this synthetic lethality extends to other components of the ESCRT machinery: *SNF8* and *VPS25* (ESCRT-II), *STP22* (ESCRT-I) and *SNF7* (ESCRT-III). Interestingly, in the yeast genetic background used in this study, loss of ESCRT components does not affect telomere length. Finally, we show that *VPS36* RNA levels are controlled by the PAF1 complex.

## Materials and methods

### Yeast strains

Standard procedures for yeast culture, mating, and tetrad dissection were followed (Adams et al. 1997). All experiments were performed using *Saccharomyces cerevisiae* W303 strains as listed in Table S1. Gene disruptions were made in diploids (DDY227, S1 Table) using one-step PCR to insert a kanMX, natMX, or hphMX cassette into the genome (Goldstein and McCusker 1999). Gene disruptions were confirmed by PCR. Oligonucleotide sequences are described in Table S2.

### Yeast growth assays

Colonies from germination plates were inoculated in liquid YEPD (supplemented with adenine) and grown until saturation overnight at 23 °C. Serial dilutions in water were spotted onto YEPD plates using a replica plating device. Plates were incubated for 2 (or 3) days at the appropriate temperatures before being photographed. Unless stated otherwise, a single plate per temperature was used (round plates typically fit between 8 and 16 strains, while rectangular plates fit between 16 and 32 strains).

### Analysis of telomere structure

Southern blot analysis was used to assess telomere length and performed as previously described. Genomic DNA was extracted, digested with XhoI, and then run overnight on a 1% agarose gel at 1 V/cm. Southern transfer was performed using the Biorad Vacuum Blotter according to the manufacturer’s indications. Y′+TG probe labelling and Southern detection were made according to the DIG High Prime DNA Labelling and Detection Starter Kit II (Roche) manufacturer’s instructions. The probe has approximately 1 kb with ~ 880 bp of Y′ and 120 bp of TG repeats, and was cut from pDL1574 using XhoI and BamHI (Dewar and Lydall 2010).

### Quantitative RT-PCR

RNA was isolated essentially as described (Collart and Oliviero 2001) followed by purification using the RNEasy Mini Kit (QIAGEN, 74,104) and DNase I digestion (Invitrogen, 18068-015). Quantitative RT-PCR was carried out using the Superscript III Platinum SYBR Green One-Step qRT-PCR kit (Invitrogen, 11736-059). RNA samples were normalized relative to the *BUD6* mRNA levels as previously described (Holstein et al. 2014).

## Results

### Neighbouring gene effect between *CDC73* and adjacent gene *VPS36*

**Fig. 2:**
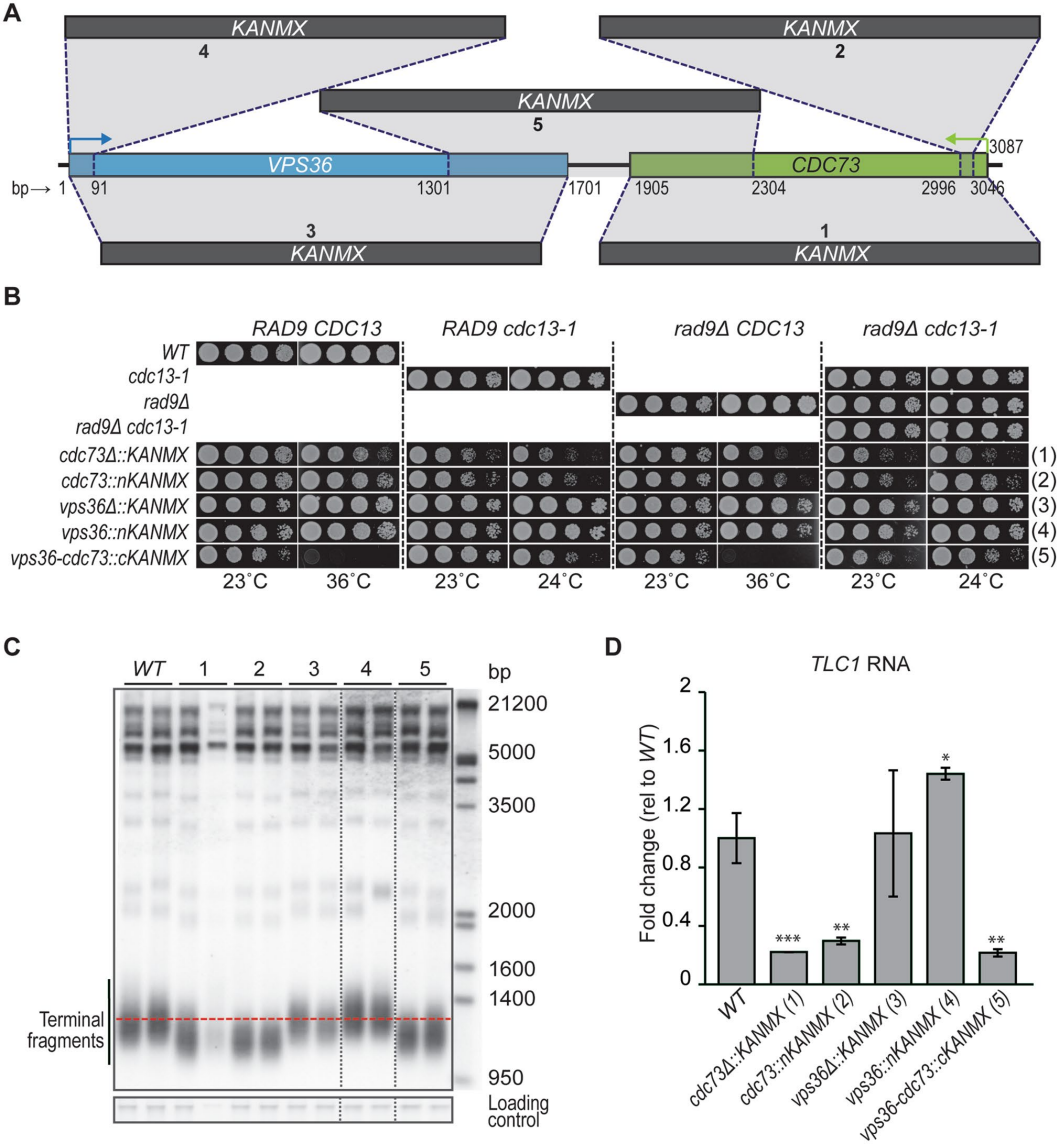
*CDC73* deletion affects *VPS36* function. **a** Map of *CDC73* and *VPS36*, together with five different constructs used for disruptions. **b** Haploid strains containing constructs in **a** combined with *CDC13 RAD9, cdc13-1 RAD9, CDC13 rad9Δ*, or *cdc13-1 rad9Δ* were grown overnight at 23 °C and serial dilutions spotted onto solid YEPD plates. Pictures were taken after 2 days of incubation at different temperatures. At each temperature, the strains were grown on the same YEPD plate. Four different strains for each genotype were analysed and a representative strain is shown (the full spot test can be seen in Fig. S1). **c** Telomeric Southern blot was performed using a Y’+TG probe to analyse the telomere structure of the strains with deletions described in **a**. Overnight cultures (grown at 23 °C) were used to isolate DNA. All lanes are from the same gel and vertical dashed lines represent the place where the gel was cut and pasted for presentation purposes. **d** RT-qPCR analysis of *TLC1* RNA levels. RNA from two independent strains was measured except for *WT* and *vps36Δ::KANMX* strains, where the two independent strains were analysed twice. Each value was normalized to the levels of *BUD6* RNA. The mean is indicated and the “error” bars indicate the two independent measurements, or average deviation. Statistical analyses used the two-tailed unpaired *T* test (*P < 0.05, **P < 0.01 and ***P < 0.001)

*CDC73* and *VPS36* point towards each other, with the stop codons separated by 204 bp (Fig. 2a), and *vps36Δ* and *cdc73Δ* have been suggested to demonstrate an adjacent gene effect (Ben-Shitrit et al. 2012). Importantly, both genes are reported to affect telomere length and the fitness of telomere defective cells (Mozdy et al. 2008; Addinall et al. 2011; Dieckmann et al. 2016). To clarify the contributions of *CDC73* and *VPS36* to the fitness of telomere defective, *cdc13-1* cells, five related gene disruption constructs were created (Fig. 2a). Constructs 1 and 3 have *CDC73* or *VPS36* ORFs completely deleted (*cdc73Δ* or *vps36Δ*); constructs 2 and 4 have interruptions in the N-termini of *CDC73* or *VPS36* ORFs (farthest from each other, therefore, less likely to affect the adjacent gene) (*cdc73Δn* or *vps36Δn*). Construct 5 has a simultaneous deletion of the C-termini of *CDC73* and VPS36 (predicted to disrupt the function of both genes) (*cdc73Δc-vps36Δc*). The effects of the constructs were analysed in *WT* (*CDC13), cdc13-1* (causing a temperature-sensitive telomere defect), *rad9Δ* (causing a defect in the DNA damage checkpoint), and *rad9Δ cdc13-1* backgrounds to determine their effects in cell fitness. Their effects on telomere length and *TLC1* RNA levels were also measured.

Importantly, and consistent with the existence of an adjacent gene effect, there is a small but consistent fitness difference between *cdc73Δ* and *cdc73Δn* strains in all combinations of *cdc13-1* and *rad9Δ* backgrounds (Fig. 2b). Overall, *cdc73Δn* cells (most likely with *VPS36* unaffected and, therefore, presumably the *CDC73* null phenotype) are fitter than *cdc73Δ* cells. As reported before, *vps36Δ cdc13-1* cells are slightly fitter than *cdc13-1* cells at 27 °C (Fig. S1B) (Dieckmann et al. 2016) and we see no detectable fitness difference between *vps36Δ* and *vps36Δn* cells in *cdc13-1* mutants (compare constructs 3 and 4, Fig. 2b). However, the strongest fitness phenotype observed was in *cdc73Δc-vps36Δc* cells at high temperatures (Fig. 2b). *cdc73Δc-vps36Δc* cells (*CDC13 RAD9*), missing both Cdc73 and Vps36, are considerably less fit than *cdc73Δ* or *cdc73Δn* cells (construct 5 *versus* 1 and 2) (Fig. 2b, 36 °C), suggesting that loss of the full *CDC73* coding sequence only partially reduces Vps36 function (in *cdc73Δ* cells). We conclude that *VPS36* and *CDC73* most likely exhibit both an adjacent gene effect and a synthetic genetic interaction.

We next measured telomere length and TLC1 RNA levels to test if the *CDC73*-*VPS36* adjacent gene effect contributed to the short telomeres of *cdc73Δ* and *vps36Δ* cells (Rog et al. 2005; Mozdy et al. 2008; Dieckmann et al. 2016). As expected, the three constructs that inactivate Cdc73 (constructs 1, 2, 5, Fig. 2a) each caused short telomeres and similarly decreased TLC1 RNA levels (Fig. 2c, d). Surprisingly, and different from previous reports, loss of Vps36 in *vps36Δ* and *vps36Δn* cells (constructs 3 and 4, Fig. 2a) did not decrease telomere length (Fig. 2c) (Askree et al. 2004; Rog et al. 2005; Dieckmann et al. 2016). If anything, *vps36Δn*, but not *vps36Δ*, slightly increased telomere length and TLC1 levels (Fig. 2c, d, constructs 3 and 4). These observations are consistent with an adjacent gene effect on telomere length and can be explained by opposing effects of Vps36 and Cdc73 on telomere length. If it is assumed that *vps36Δn* causes the null phenotype (increased TLC1 and telomere length), then full-length deletion, *vps36Δ*, reduces *CDC73* function counteracting the increase in TLC1 (telomere length).

The effect of Vps36 on TLC1 RNA levels is not shared with other ESCRT components (Fig. S2), suggesting this is a specific function of Vps36. Overall, telomere length and TLC1 levels correlate with each other, but not with the fitness defects, across the different deletions (Fig. 2c, d versus Fig. 2b). For instance, *cdc73Δc-vps36Δc* cells show the strongest fitness defects, but, from the strains missing Cdc73 (*cdc73Δc-vps36Δc, cdc73Δ* or *cdc73Δn*), are perhaps the ones with the longest telomeres (Fig. 2b, c, constructs 1 and 2 versus construct 5). We conclude that the adjacent gene effect does not contribute to Cdc73 regulation of TLC1 RNA and telomere shortening is not the reason for *cdc73-vps36Δc* synthetic fitness defects.

Overall, our data suggest that the standard *cdc73Δ* mutation (construct 1) is partially interfering with *VPS36* activity, most likely by affecting the *VPS36* 3′UTR. Furthermore, analysis of telomere lengths suggests that the standard *VPS36* deletion affects *CDC73* function. These observations confirm the existence of an adjacent gene effect between *CDC73* and *VPS36*. We also report a synthetic fitness defect in cells simultaneously depleted of Vps36 and Cdc73, which is likely independent of the telomere length of these cells.

### The PAF1 complex regulates *VPS36* transcription

**Fig. 3:**
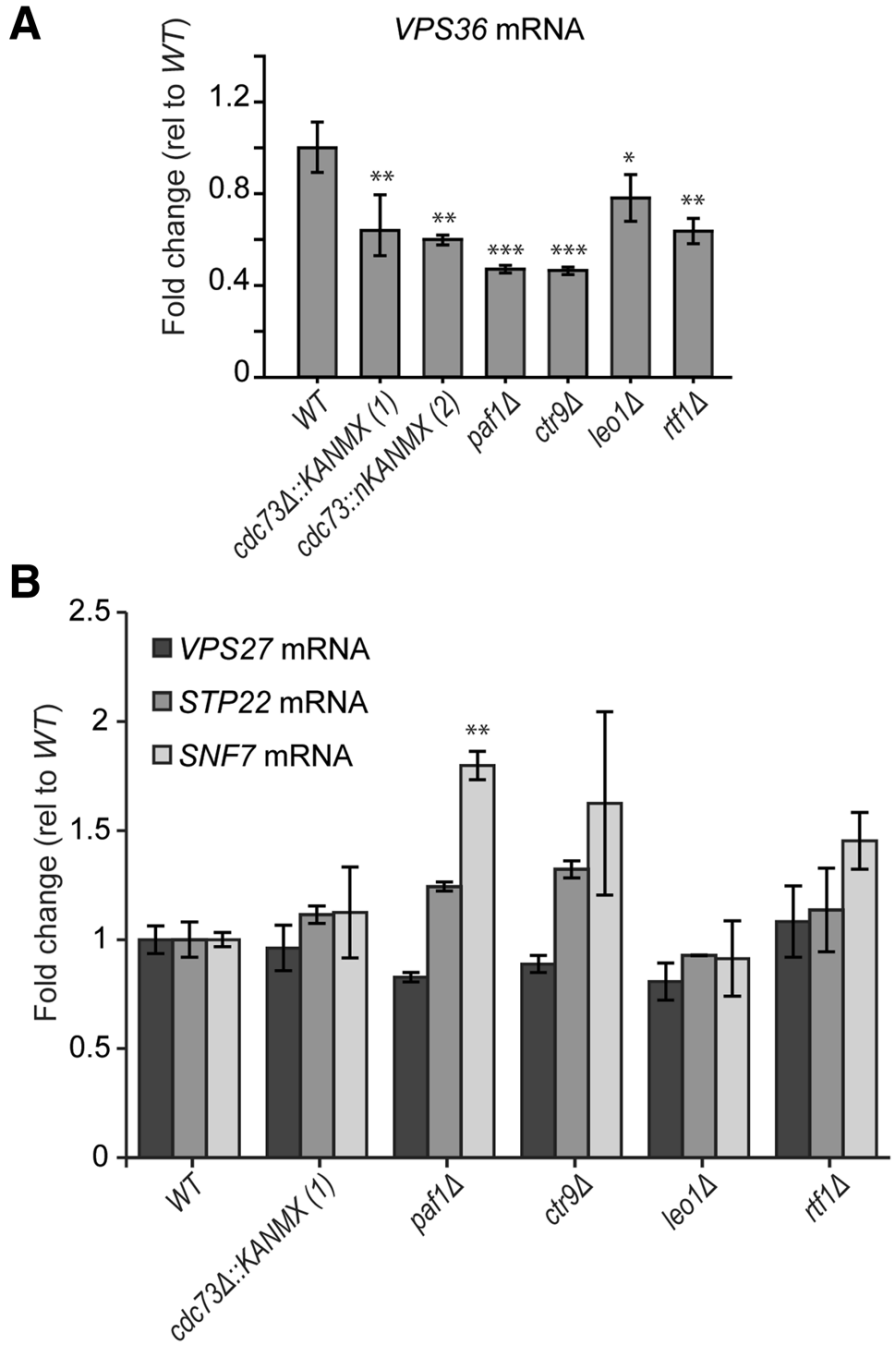
PAF1 complex controls VPS36 expression. **a, b** RT-qPCR analysis of *VPS36* (**a**), *STP22* (**b**), *VPS27* (**b**) and *SNF7* (**b**) RNA levels. RNA from two independent strains was measured as described in Fig. 2d. Statistical analyses used the two-tailed unpaired *T* test (*P < 0.05, **P < 0.01 and ***P < 0.001)

To test if the knockout of *CDC73* was affecting *VPS36* transcription and/or RNA stability, *VPS36* RNA levels were measured in strains carrying the two disruptions of *CDC73* (constructs 1 and 2, Fig. 2a). *VPS36* RNA was also measured in strains with the other PAF1 components deleted, since the PAF1 complex is involved in transcriptional regulation and altered *VPS36* RNA levels in *cdc73Δ* cells could be partially due to defects in the PAF1 complex activity. Interestingly, *VPS36* RNA is decreased when each of the PAF1 complex components is deleted, with little difference between deletion or N-termini disruption of *CDC73* (constructs 1 and 2, Fig. 3a). The lack of difference between *cdc73Δ* and *cdc73Δn* suggests that the adjacent gene effect does not affect *VPS36* RNA levels, but, perhaps, an altered *VPS36* 3′-UTR could affect translation and/or mRNA localization. Finally, to test if all ESCRT machinery is under PAF1-dependent transcriptional regulation, the RNA levels of other ESCRT components were next analysed in PAF1 complex mutants. *VPS27* (ESCRT-0), *STP22* (ESCRT-I) and *SNF7* (ESCRT-III) RNA levels were not reduced by deletion of any of the PAF1 complex components (Fig. 3b). In fact, *SNF7* mRNA levels were significantly increased in *paf1Δ* mutants (and probably in *ctr9Δ* mutants). This is unusual, because *paf1Δ* decreases levels of most transcripts (Xu et al. 2017).

We conclude that the adjacent gene effect between *CDC73* and *VPS36* does not affect *VPS36* RNA levels, but could, for instance, affect VPS36 mRNA localization and/or translation. In addition, we show that *VPS36* RNA is regulated by the PAF1 complex.

### ESCRT complex does not affect telomere length of *S. cerevisiae* W303 cells

**Fig. 4:**
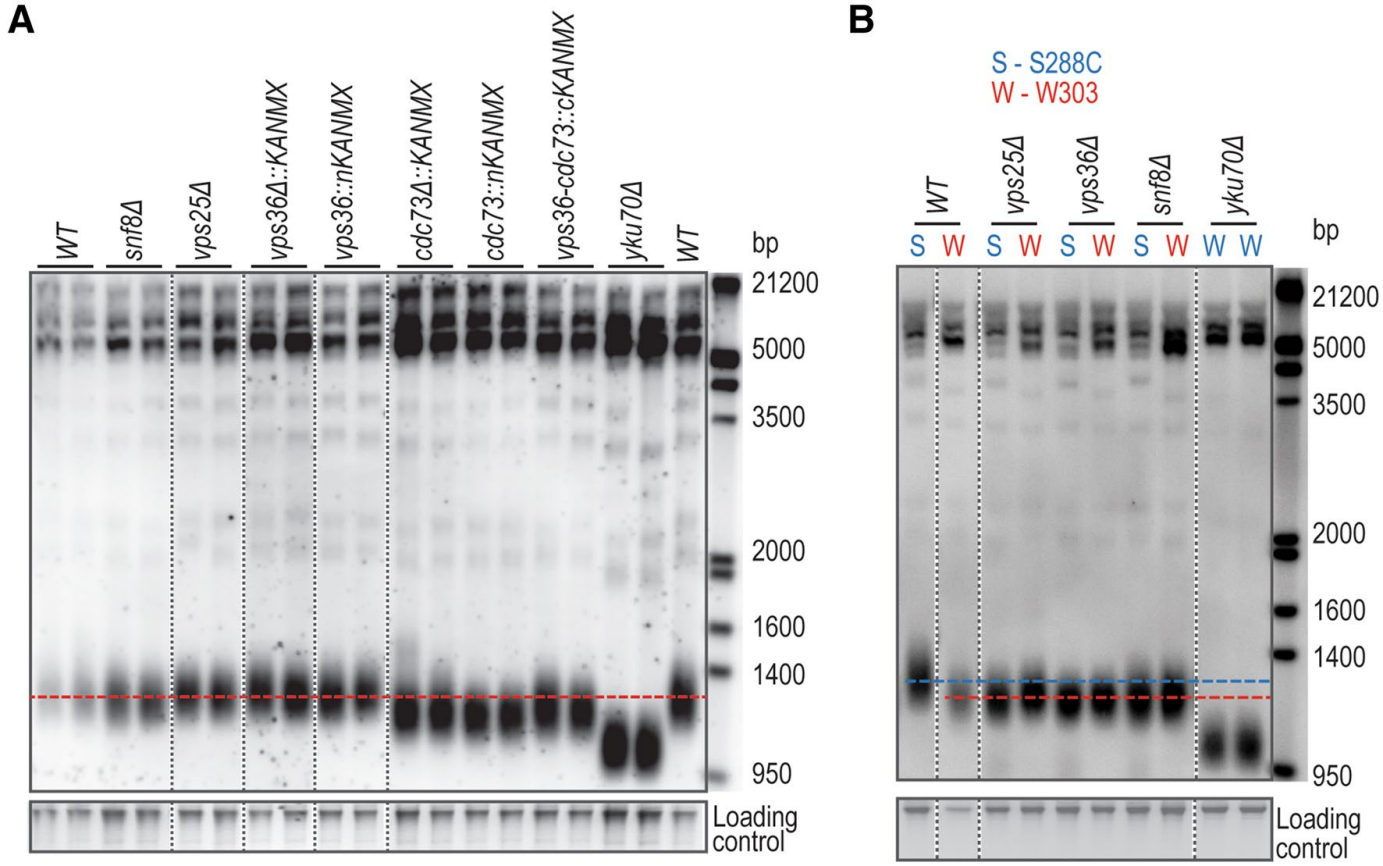
ESCRT-II mutants have normal telomeres in *S. cerevisiae* W303 cells. **a** Southern blot as described in Fig. 2c. **b** Telomere southern blot of *WT, vps25Δ, vps36Δ* and *snf8Δ* mutations in S288C and W303 genetic backgrounds. S288C strains are from the deletion database. Southern blot performed as described in Fig. 2c, except that the cells were cultured at 30 °C. In each panel, all lanes are from the same gel and vertical dashed lines represent the place where the gel was cut and pasted for presentation purposes

The absence of a telomere length phenotype in *vps36Δ* cells observed in Fig. 2c is not in agreement with the previously reported short telomeres observed in *ESCRT* mutants (Askree et al. 2004; Rog et al. 2005; Dieckmann et al. 2016). Therefore, we asked if normal telomere length was also observed in cells with mutations in other components of the ESCRT-II complex. To test this, the telomere length in *snf8Δ* or *vps25Δ* strains, defective in other components of the ESCRT-II complex, was measured. Interestingly, no strong effects on telomere length were observed upon deletion of any of the ESCRT-II complex components (Fig. 4a). On the other hand, as expected, *CDC73* deletion or N-terminal disruption and *YKU70* deletion led to shorter telomeres (Fig. 4a). Our experiments were performed in the W303 genetic background, and cells were cultured at 23 °C, whereas the published experiments were in the BY4742 (S288C) background, and cells were most likely cultured at 30 °C. To test if telomere length in *ESCRT* mutants is particularly sensitive to temperature, the telomere length of the same mutants analysed in Fig. 2 was measured after growth at 30 °C. There was still no observable telomere length decrease in any of the ESCRT complex mutants cultivated at 30 °C (Fig. S3). We next compared the telomere length of *WT, vps25Δ, vps36Δ*, and *snf8Δ* cells in the W303 and S288C genetic backgrounds (at 30 °C). As previously reported, *WT* cells of the S288C genetic background have longer telomeres than W303 cells (Fig. 4b) (Lebel et al. 2009). Interestingly, when compared to *WT* cells, the telomeres of the ESCRT mutants are relatively short in the S288C background but relatively normal in the W303 background (Fig. 4b). We conclude that the previously reported telomere length regulation by the ESCRT machinery is not conserved in *S. cerevisiae* W303 cells and is most likely strain background-dependent.

### *vps36Δ* is synthetically lethal with *paf1Δ* or *ctr9Δ* but not with *cdc73Δ, rtf1Δ* or *leo1Δ*

**Fig. 5:**
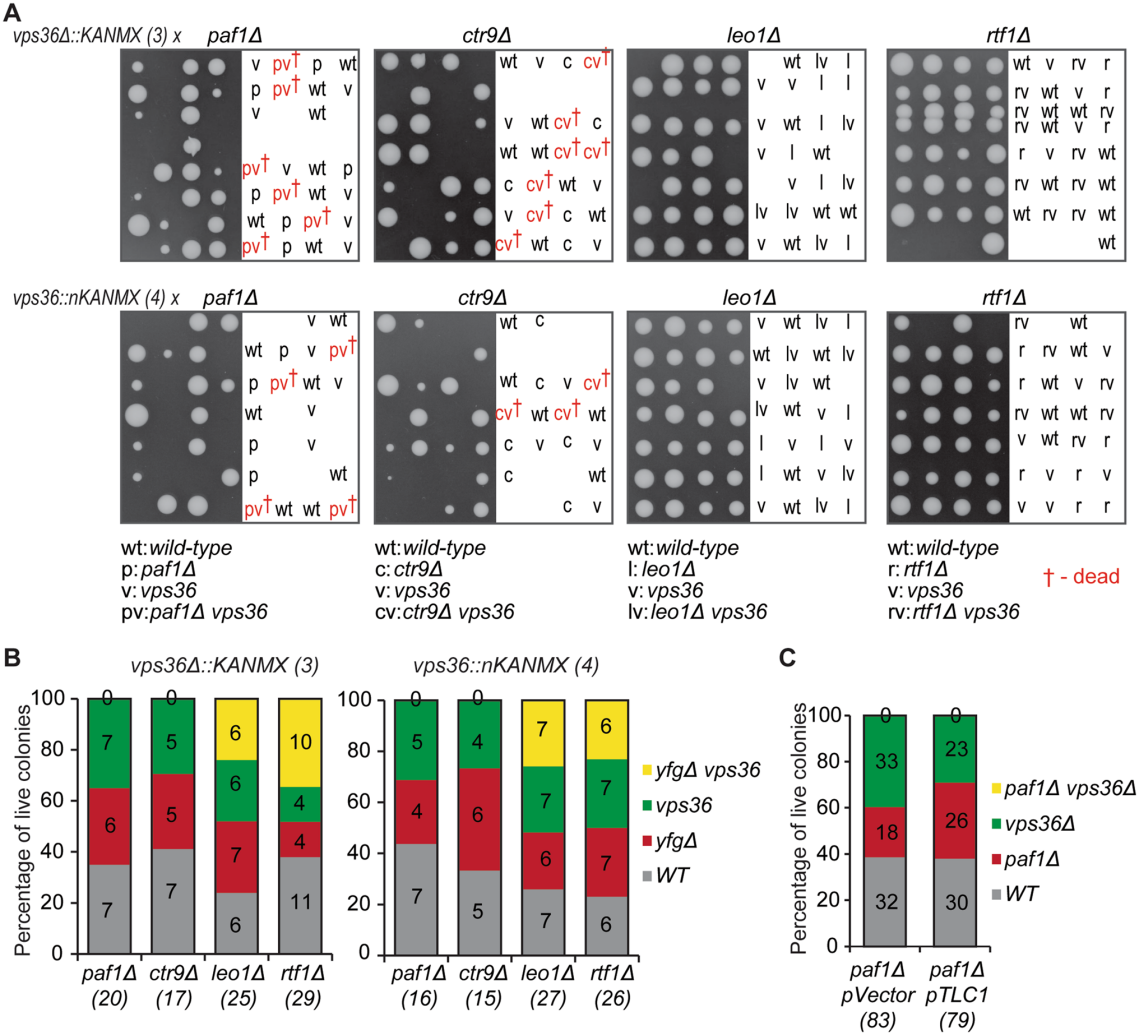
Vps36 is needed for the viability of *paf1Δ* or *ctr9Δ* but not *rtf1Δ* or *leo1Δ* cells. **a** *vps36Δ* or *vps36Δn* (Fig. 2a, constructs 3 and 4) were crossed to *paf1Δ, ctr9Δ, rtf1Δ*, or *leo1Δ* strains and diploid cells were obtained. Diploids were sporulated and tetrads dissected onto YEPD plates. Germinated spores grew for 6 days at 23 °C before being photographed. Genotypes of each spore are indicated on the right when possible. Genotype of inviable spores was inferred using the expected 2:2 Mendelian segregation. **b** Quantification of A. Percentage of colonies of each genotype is shown (relative to the total number of viable colonies). The total number of colonies genotyped are shown in brackets. **c** A *vps36Δ::KANMX paf1Δ* heterozygous diploid was transformed with a centromeric plasmid carrying *TLC1* (pDL751) or a vector plasmid (pDL1713). Random spore analysis was used to obtain haploid strains: the diploids carrying the plasmids were sporulated and plated onto –URA plates. After 7 days at 23 °C, colonies were genotyped. The percentage of each genotype was plotted as in **b**

Given that *VPS36* and *CDC73* contribute independently to maintain cell fitness, particularly at high temperature, we wanted to know what role, if any, *VPS36* played in the fitness of other PAF1 complex deletion strains. To address this question, we crossed *vps36Δ* and *vps36Δn* (Fig. 2a, disruptions 3 and 4) to strains carrying *paf1Δ, ctr9Δ, rtf1Δ*, or *leo1Δ* mutations. Note that fitness of *cdc73Δ vps36Δ* double mutants was previously tested and found to be decreased when compared to single mutants (Fig. 2b). Interestingly, *vps36Δ paf1Δ* or *vps36Δ ctr9Δ* double mutants could not be identified, but all other double mutants were viable (Fig. 5a, quantification in Fig. 5b). Although this synthetic lethality between *vps36Δ* and *paf1Δ* or *ctr9Δ* has not been previously reported, both *PAF1* and *CTR9* deletions were reported to show synthetic growth defects with deletions affecting ubiquitylation pathways (*UBP6*) or multivesicular body/protein sorting pathways (*VPS21, VPS71*, and *VPS72*) (Krogan et al. 2003b; Laribee et al. 2005; Collins et al. 2007; Hang et al. 2011). Since Vps36 (as a component of ESCRT-II) is involved in sorting ubiquitylated proteins for degradation through the multivesicular body pathway, the findings here reported are in line with the previously described genetic interactions of *PAF1*/*CTR9* and members of the multivesicular body degradation pathway (Krogan et al. 2003b; Laribee et al. 2005; Collins et al. 2007; Hang et al. 2011). Together, all these results show that Vps36 and the PAF1 complex, especially Paf1 and Ctr9, work in independent pathways to support cell viability.

*CDC73, PAF1*, and *CTR9* deletions decrease *TLC1* RNA levels, leading to short telomeres and growth defects (Mozdy et al. 2008). Therefore, we asked if the synthetic sickness/lethality between PAF1 and ESCRT mutants was caused by a TLC1-dependent mechanism. If so, then overexpression of *TLC1* in *vps36Δ paf1Δ* mutants may render these cells more viable. To test this hypothesis, *vps36Δ paf1Δ* heterozygous diploids were transformed with a 2 µm TLC1 plasmid and the presence of viable *vps36Δ paf1Δ* (pTLC1) haploid progeny was assessed after sporulation. However, there was no effect of *TLC1* plasmid (versus vector) on the viability of *vps36Δ paf1Δ* (from 79 viable spores carrying *TLC1*, none was *vps36Δ paf1Δ*) (Fig. 5c).

We conclude that Vps36 and Paf1/Ctr9 work in independent pathways to maintain cell viability, with simultaneous lack of Vps36 and Paf1 or Ctr9 leading to cell death. Furthermore, the synthetic lethality between *vps36Δ* and *paf1Δ*/*ctr9Δ* is unlikely to be related to the decreased levels of TLC1 in *paf1Δ* cells.

### *paf1Δ* and *ctr9Δ* are synthetically lethal with deletions of ESCRT-I, ESCRT-II and ESCRT-III components

**Fig. 6:**
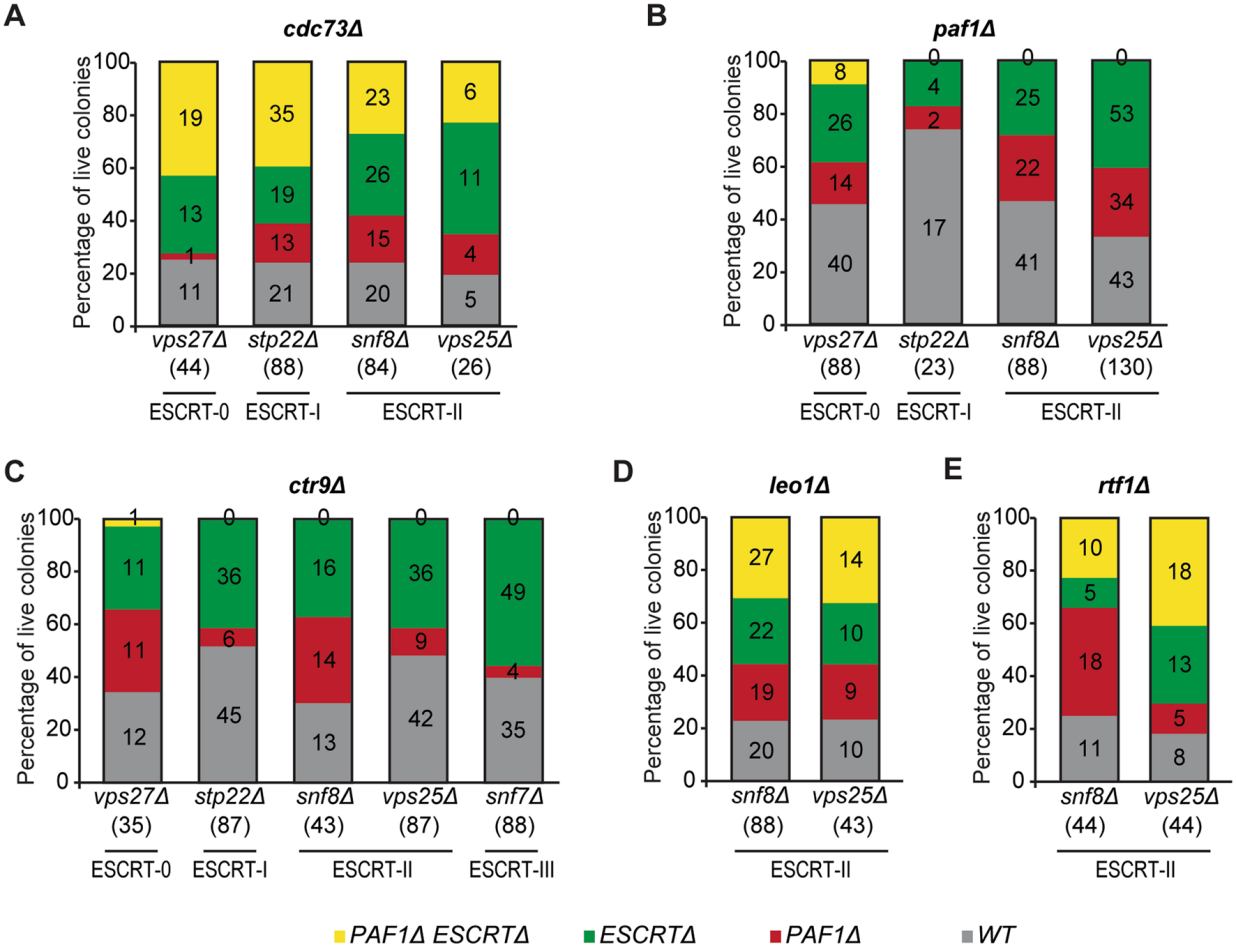
*paf1Δ* and *ctr9Δ* are synthetically lethal with deletions affecting ESCRT-I, ESCRT-II and ESCRT-III components. **a**–**e** Strains carrying deletions of different components of the ESCRT machinery complex were crossed with *cdc73Δ* (**a**), *paf1Δ* (**b**), *ctr9Δ* (**c**), *leo1Δ* (**d**), or *rtf1Δ* (**e**) strains. Data were analysed as described in Fig. 5b

To test if the synthetic lethality between *paf1Δ*/*ctr9Δ* and *vps36Δ* is conserved with other ESCRT components, we analysed the progeny of diploids heterozygous for deletions of PAF1 complex components and *VPS27* (ESCRT-0), *STP22* (ESCRT-I), *SNF8* (ESCRT-II), *VPS25* (ESCRT-II), and *SNF7* (ESCRT-III) (Fig. 1). Interestingly, and in agreement with the interactions observed between *PAF1*/*CTR9* and *VPS36, PAF1* and *CTR9* deletion in cells carrying deletions of any of the tested ESCRT-I to -III components led to synthetic lethality (Fig. 6). In most cases, the double mutants divided 3–5 times before stopping divisions, as measured by micro-colony analysis (Fig. S4). The fact that these cells can divide a number of times before death suggests that they accumulate defects that eventually lead to cell death. Interestingly, 8 (of 88) *paf1Δ vps27Δ* and 1 (of 35) *ctr9Δ vps27Δ* cells were viable, suggesting that Vps27 (ESCRT-0) is not as important as downstream ESCRT components for the viability of *paf1Δ* or *ctr9Δ* cells. We conclude that PAF1 complex (through Paf1 and Ctr9) works with the ESCRT machinery, in independent pathways, to maintain cell viability.

## Discussion

Budding yeast is a powerful model organism to systematically study eukaryotic gene function and interactions, with many gene functions being conserved in human cells. For instance, yeast genome-wide screens have been used extensively for analysis and discovery of new gene interactions in various fields: transcription elongation and chromatin modification (Krogan et al. 2003b), DNA replication (Dubarry et al. 2015), and telomere length maintenance (TLM) (Ungar et al. 2009). However, there are caveats associated with the use of systematic yeast genetic approaches. For instance, the deletion of one gene ORF might affect its adjacent gene (adjacent gene effect). Indeed, it has been suggested that 10% of all budding yeast genes demonstrate an adjacent gene effect (Ben-Shitrit et al. 2012; Atias et al. 2016). Another issue is related to the genetic backgrounds used. Since the principal yeast deletion library was created in the S288C genetic background, most of the genome-wide data available is in this background (Giaever et al. 2002). However, other commonly used yeast genetic backgrounds like W303, SK1, Y55, among others, show important differences, such as telomere length (Lebel et al. 2009). Thus, it is important to test for the role of adjacent gene effects and of genetic backgrounds.

*CDC73* encodes a component of the PAF1 complex, involved in transcription regulation, and is adjacent to *VPS36*, encoding a component of the ESCRT machinery, involved in membrane remodelling (mainly to sort ubiquitylated proteins to the vacuole). Bioinformatic analysis identified *CDC73* and *VPS36* as potentially demonstrating an adjacent gene effect, since deletion of either causes short telomeres and the ORFs are only separated by 204 bp (Ben-Shitrit et al. 2012). On the other hand, proteins that work with Cdc73 in the PAF1 complex, and with Vps36 in the ESCRT machinery also affect telomere length, suggesting that there may not be an adjacent gene effect on telomere length.

To clarify whether there is an adjacent gene effect between *CDC73* and *VPS36* we compared the fitness and telomere lengths of cells with different *CDC73* and *VPS36* ORF disruptions. We established that complete *CDC73* deletion partially reduces *VPS36* function to affect fitness. Since we did not see any change in *VPS36* mRNA levels in strains with *CDC73* ORF deletion or an N-terminal disruption, it is likely that *CDC73* ORF deletion affects *VPS36* mRNA localization and/or translation. Complete *VPS36* deletion partially reduces *CDC73* function to affect telomere length (without noticeable effects on fitness). We conclude that *CDC73* and *VPS36* show somewhat complex adjacent gene effects.

While investigating the adjacent gene effect between *CDC73* and *VPS36*, we noted that in the W303 yeast genetic background, *ESCRT* mutants do not affect telomere length, in contrast to what has been reported for the S288C yeast genetic background where *ESCRT* mutations caused short telomeres (Rog et al. 2005; Ungar et al. 2009; Dieckmann et al. 2016). This difference shows that the relatively well studied role of *ESCRT* mutants in telomere length is not conserved within different *S. cerevisiae* genetic backgrounds; therefore, it might not be preserved in other species (like mammals) (Rog et al. 2005; Dieckmann et al. 2016). However, it is possible that ESCRT genes affect telomere function in both S288C and W303, but that it is only possible to see an effect on telomere length when telomeres are long (S288C), rather than short (W303). Thus, more work is needed to understand the true role of the ESCRT machinery in telomere biology.

In addition to the adjacent gene effect, we describe other interactions between the PAF1 complex (composed of Cdc73, Paf1, Ctr9, Leo1, and Rtf1) and *VPS36* (ESCRT machinery). First, we report that all components of the PAF1 complex regulate *VPS36* RNA levels. In addition, we found that *vps36Δ* exacerbates the temperature sensitivity of *cdc73Δ* mutants. Furthermore, there is a synthetic lethal interaction in cells with defective ESCRT-I (*stp22Δ*), ESCRT-II (*vps36Δ, snf8Δ* and *vps25Δ*), and ESCRT-III (*snf7Δ*) machineries and *ctr9Δ* or *paf1Δ* mutations. *LEO1* and *RFT1* do not show synthetic genetic interactions with genes encoding ESCRT components, in agreement with their minor role in the PAF1 complex integrity (Jaehning 2010; Tomson and Arndt 2013; Rodrigues and Lydall 2018). These show that the ESCRT machinery functions redundantly with Ctr9/Paf1 to maintain yeast cell viability.

How the PAF1 complex and the ESCRT machinery function together to maintain yeast cell fitness is not yet clear. In yeast, ESCRT-0 is as important as the other ESCRT complexes (-I, -II, and -III) in the MVB formation (to degrade ubiquitylated cargo) (Schmidt and Teis 2012). Therefore, the fact that *paf1Δ*/*ctr9Δ* are not synthetically lethal with *vps27Δ* (ESCRT-0) suggests that defective MVB pathway is not the major cause for the synthetic lethality of *paf1Δ*/*ctr9Δ* with ESCRT-I, ESCRT-II, and ESCRT-III mutants. *PAF1* and *CTR9* deletions lead to severe alterations in the transcriptome, which is believed to be the cause of the strong fitness defects of these mutants (Xu et al. 2017; Rodrigues and Lydall 2018). The higher sensitivity of ESCRT deletion mutants to 6-azauracil and the fact that ESCRT proteins associate with 3′ regions of actively transcribed genes suggested that the ESCRT components could also play a role in transcription elongation (Song et al. 2014). Thus, *paf1Δ vpsΔ* and *ctr9Δ vpsΔ* might have worse transcriptional defects leading to cell death. Supporting the idea that the ESCRT machinery is important in cells with transcription defects, histone deacetylases (*HDA1, HDA2*, and *HDA3*), and mRNA surveillance genes (*PAP2*) were found to be essential in *snf8Δ, stp22Δ, vps25Δ*, and *vps36Δ* cells (Pan et al. 2006; Lin et al. 2008).

In conclusion, we report that the PAF1 complex and the ESCRT machinery work together to maintain the fitness of W303 yeast cells. We confirm that deletion of *CDC73* affects *VPS36* and vice versa. Therefore, these particular adjacent genes, encoding PAF1 and ESCRT machinery components, interact both in *cis* and in *trans*. Thus, these studies indicate how complex it can be to interpret the effects of single gene deletions in budding yeast, and have implications for systematic studies in all cell types. Finally, we show that ESCRT mutations do not affect the telomere length of W303 yeast cells, while confirming that they decrease the telomere length of S288C yeast cells.

## Electronic supplementary material

Below is the link to the electronic supplementary material.

Supplementary material 1 (PDF 773 KB)
