# Supplementary material for: Cis and trans interactions between genes encoding PAF1 complex and ESCRT machinery components in yeast
Source: Curr Genet. 2018 Mar 22;64(5):1105–16. doi: 10.1007/s00294-018-0828-6 (PMC6153643; doi:10.1007/s00294-018-0828-6)
Supplement: Supplementary file 1 — Supplementary material 1 (PDF 773 KB) [file 294_2018_828_MOESM1_ESM.pdf]

Supplemental Information

Cis and *trans* interactions between genes encoding PAF1 complex and ESCRT machinery components in yeast

Joana Rodrigues and David Lydall*

Institute for Cell and Molecular Biosciences, Newcastle University Medical School, Newcastle upon Tyne, United Kingdom

* Corresponding author
E-mail: David.Lydall@newcastle.ac.uk
Tel: +44 (0) 191 208 5318

This file contains:
Fig. S1 *CDC73* and its adjacent gene *VPS36* interact.

Fig. S2 Deletion of ESCRT components does not affect TLC1 levels.

Fig. S3 *S. cerevisiae* W303 cells ESCRT-II mutants have normal telomeres.

Fig. S4 Inviable double mutants (*PAF1 ESCRT*) divide a few times before arresting.

Table S1 Yeast strains used in this study.

Table S2 List of primers used.

Fig. S1. *CDC73* and its adjacent gene *VPS36* interact.

(A-D) Complete spot test as described in Fig. 2B.

Fig. S2. Deletion of ESCRT components does not affect TLC1 levels.

RT-qPCR analysis of *TLC1* RNA levels. RNA from two independent strains was measured as described in Fig. 2D. Statistical analyses used the two-tailed unpaired T test (no significant differences were observed).

Fig. S3 *S. cerevisiae* W303 cells ESCRT-II mutants have normal telomeres.

Southern blot as described in Fig. 2C. Cells were cultivated at 30°C for 3 passages (each passage corresponds to 2 days growing in YEPD plates) before they were inoculated in liquid YEPD and grown overnight at 30°C. DNA was isolated from the saturated liquid cultures. On the right panel are the same samples shown in Fig. 4A. Vertical dashed line was added for presentation purposes to help indicate the different culture temperatures.

Fig. S4. Inviable double mutants (*PAF1 ESCRT*) divide a few times before arresting.

(A-C) Heterozygous diploids carrying PAF1 complex and ESCRT complex deletions were analysed as in Fig. 5A. Microcolony pictures of the double mutants were taken after 3 days (40X objective) at 23°C and reproduced at the same scale. **(D)** Comparative size of the microcolonies originated from cells with different genotypes.

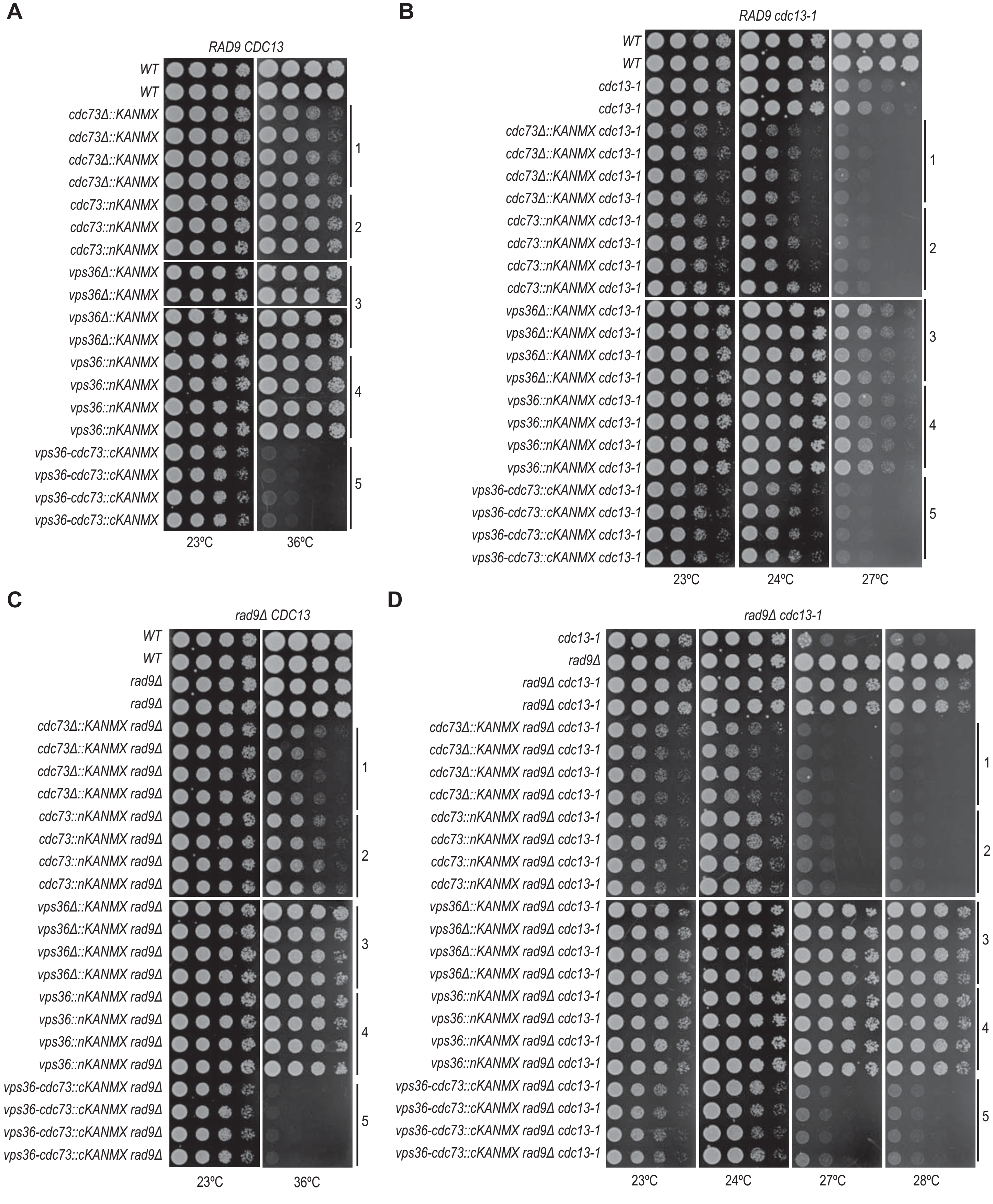
A
RAD9 CDC13
WT
WT
cdc73Δ::KANMX
cdc73Δ::KANMX
cdc73Δ::KANMX
cdc73Δ::KANMX
cdc73::nKANMX
cdc73::nKANMX
cdc73::nKANMX
vps36Δ::KANMX
vps36Δ::KANMX
vps36Δ::KANMX
vps36Δ::KANMX
vps36::nKANMX
vps36::nKANMX
vps36::nKANMX
vps36::nKANMX
vps36-cdc73::cKANMX
vps36-cdc73::cKANMX
vps36-cdc73::cKANMX
vps36-cdc73::cKANMX
1
2
3
4
5
23ºC
36ºC
B
RAD9 cdc13-1
WT
WT
cdc13-1
cdc13-1
cdc73Δ::KANMX cdc13-1
cdc73Δ::KANMX cdc13-1
cdc73Δ::KANMX cdc13-1
cdc73Δ::KANMX cdc13-1
cdc73::nKANMX cdc13-1
cdc73::nKANMX cdc13-1
cdc73::nKANMX cdc13-1
cdc73::nKANMX cdc13-1
vps36Δ::KANMX cdc13-1
vps36Δ::KANMX cdc13-1
vps36Δ::KANMX cdc13-1
vps36Δ::KANMX cdc13-1
vps36::nKANMX cdc13-1
vps36::nKANMX cdc13-1
vps36::nKANMX cdc13-1
vps36::nKANMX cdc13-1
vps36-cdc73::cKANMX cdc13-1
vps36-cdc73::cKANMX cdc13-1
vps36-cdc73::cKANMX cdc13-1
vps36-cdc73::cKANMX cdc13-1
1
2
3
4
5
23ºC
24ºC
27ºC
C
rad9Δ CDC13
WT
WT
rad9Δ
rad9Δ
cdc73Δ::KANMX rad9Δ
cdc73Δ::KANMX rad9Δ
cdc73Δ::KANMX rad9Δ
cdc73Δ::KANMX rad9Δ
cdc73::nKANMX rad9Δ
cdc73::nKANMX rad9Δ
cdc73::nKANMX rad9Δ
cdc73::nKANMX rad9Δ
vps36Δ::KANMX rad9Δ
vps36Δ::KANMX rad9Δ
vps36Δ::KANMX rad9Δ
vps36Δ::KANMX rad9Δ
vps36::nKANMX rad9Δ
vps36::nKANMX rad9Δ
vps36::nKANMX rad9Δ
vps36::nKANMX rad9Δ
vps36-cdc73::cKANMX rad9Δ
vps36-cdc73::cKANMX rad9Δ
vps36-cdc73::cKANMX rad9Δ
vps36-cdc73::cKANMX rad9Δ
1
2
3
4
5
23ºC
36ºC
D
rad9Δ cdc13-1
cdc13-1
rad9Δ
rad9Δ cdc13-1
rad9Δ cdc13-1
cdc73Δ::KANMX rad9Δ cdc13-1
cdc73Δ::KANMX rad9Δ cdc13-1
cdc73Δ::KANMX rad9Δ cdc13-1
cdc73Δ::KANMX rad9Δ cdc13-1
cdc73::nKANMX rad9Δ cdc13-1
cdc73::nKANMX rad9Δ cdc13-1
cdc73::nKANMX rad9Δ cdc13-1
cdc73::nKANMX rad9Δ cdc13-1
vps36Δ::KANMX rad9Δ cdc13-1
vps36Δ::KANMX rad9Δ cdc13-1
vps36Δ::KANMX rad9Δ cdc13-1
vps36Δ::KANMX rad9Δ cdc13-1
vps36::nKANMX rad9Δ cdc13-1
vps36::nKANMX rad9Δ cdc13-1
vps36::nKANMX rad9Δ cdc13-1
vps36::nKANMX rad9Δ cdc13-1
vps36-cdc73::cKANMX rad9Δ cdc13-1
vps36-cdc73::cKANMX rad9Δ cdc13-1
vps36-cdc73::cKANMX rad9Δ cdc13-1
vps36-cdc73::cKANMX rad9Δ cdc13-1
1
2
3
4
5
23ºC
24ºC
27ºC
28ºC

Figure S1

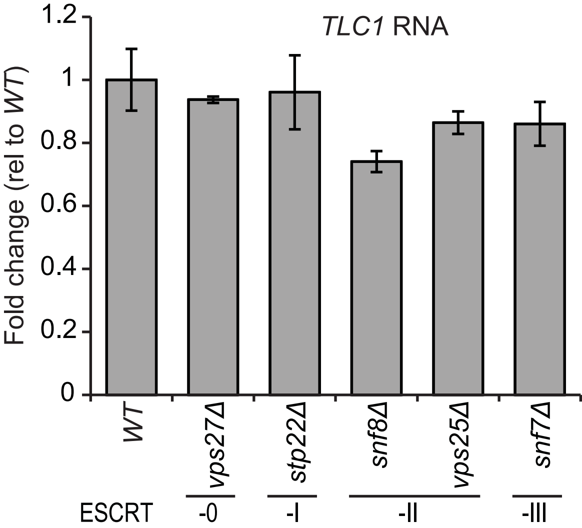

TLC1 RNA
Fold change (rel to WT)
1.2
1
0.8
0.6
0.4
0.2
0
WT
vps27Δ
stp22Δ
snf8Δ
vps25Δ
snf7Δ
ESCRT
-0
-I
-II
-III

Figure S2

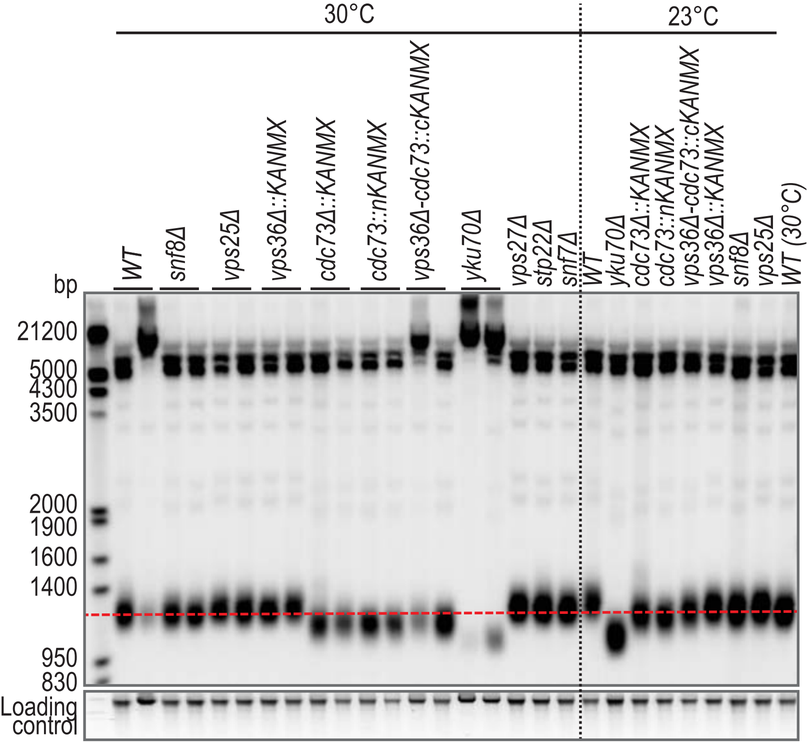
30°C
23°C
bp
21200
5000
4300
3500
2000
1900
1600
1400
950
830
Loading control
WT
snf8Δ
vps25Δ
vps36Δ::KANMX
cdc73Δ::KANMX
cdc73::nKANMX
vps36Δ-cdc73::cKANMX
yku70Δ
vps27Δ
stp22Δ
snf7Δ
WT
yku70Δ
cdc73Δ::KANMX
cdc73::nKANMX
vps36Δ-cdc73::cKANMX
vps36Δ::KANMX
snf8Δ
vps25Δ
WT (30°C)

Figure S3

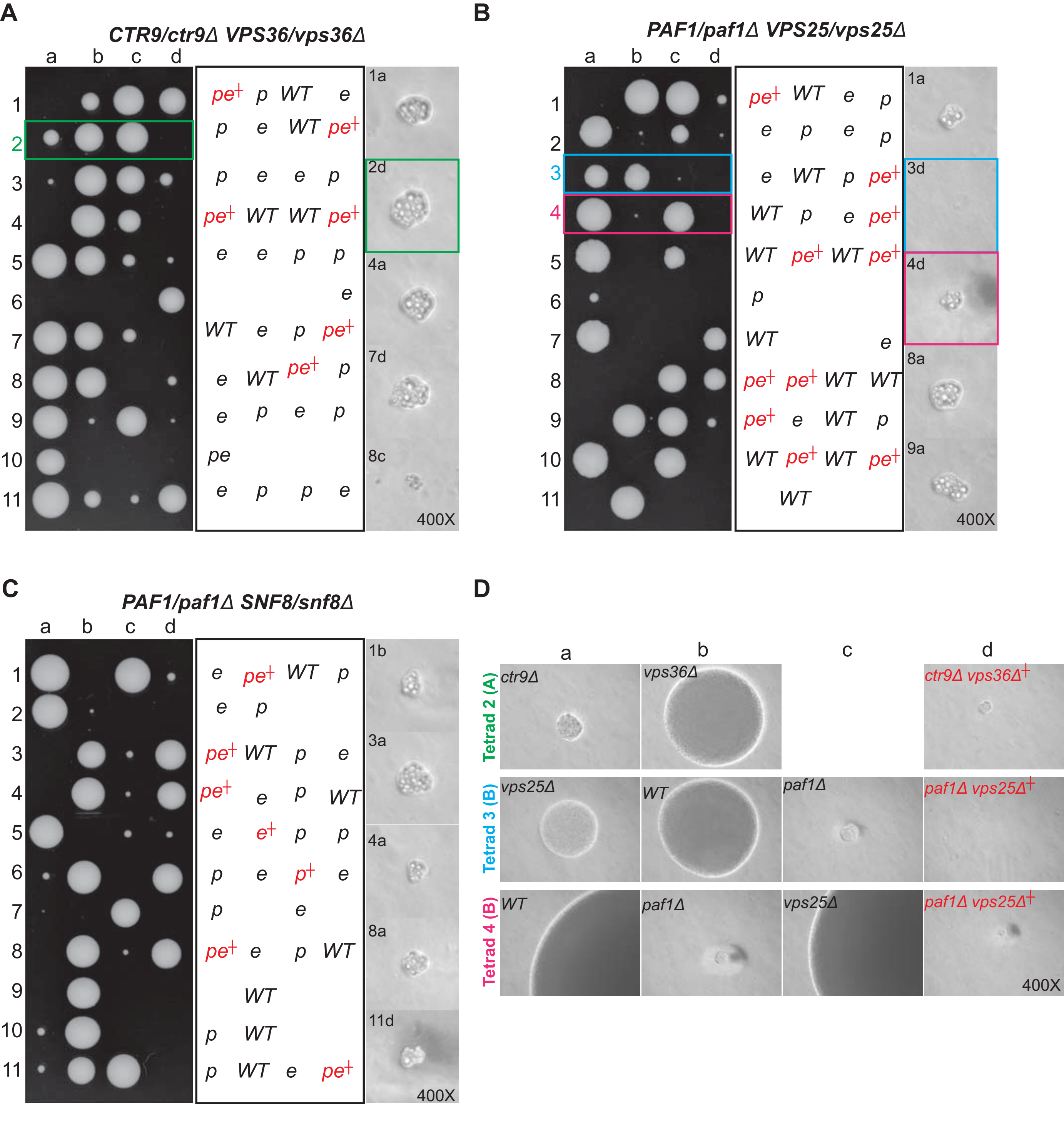

+ - *dead*
p - PAF1 complex component *(paf1Δ* or *ctr9Δ)*
e - ESCRT complex component *(snf8Δ, vps25Δ or vps36Δ)*

Figure S4

Table S1- List of the W303 strains used in this study. S288C strains (Figure 4B) were obtained from the yeast knockout library and are not described in this table.

**DLY**	**Genotype**	**Ref.**
1366	*MATα ade2-1 trp1-1 can1-100 leu2-3,112 his3-11,15 ura3 GAL+ psi+ ssd1-d2 RAD5 yku70::HIS3*	Lydall lab
1412	*MATa ade2-1 trp1-1 can1-100 leu2-3,112 his3-11,15 ura3 GAL+ psi+ ssd1-d2 RAD5 yku70::HIS3*	Lydall lab
1845	*MATa ade2-1 trp1-1 can1-100 leu2-3,112 his3-11,15 ura3 GAL+ psi+ ssd1-d2 yku70::HIS3 mre11::hisG::URA3 RAD5*	Lydall lab
1846	*MATα ade2-1 trp1-1 can1-100 leu2-3,112 his3-11,15 ura3 GAL+ psi+ ssd1-d2 yku70::HIS3 mre11::hisG::URA3RAD5*	Lydall lab
2146	*Mata tlc1::HIS3 ade2-1 t can1-100 ura3 GAL+ psi+ ssd1-d2 RAD5*	Lydall lab
2147	*Matα tlc1::HIS3 ade2-1 t can1-100 ura3 GAL+ psi+ ssd1-d2 RAD5*	Lydall lab
3001	*MATα ade2-1 trp1-1 can1-100 leu2-3,112 his3-11,15 ura3 GAL+ psi+ ssd1-d2 RAD5*	Rodney Rothstein
8460	*MATa ade2-1 trp1-1 can1-100 leu2-3,112 his3-11,15 ura3 GAL+ psi+ ssd1-d2 RAD5*	Rodney Rothstein
8490	*MATa ade2-1 trp1-1 can1-100 leu2-3,112 his3-11,15 ura3 GAL+ psi+ ssd1-d2 RAD5 cdc73::KANMX*	This study
8491	*MATα ade2-1 trp1-1 can1-100 leu2-3,112 his3-11,15 ura3 GAL+ psi+ ssd1-d2 RAD5 cdc73::KANMX*	This study
8736	*MATa ade2-1 trp1-1 can1-100 leu2-3,112 his3-11,15 ura3 GAL+ psi+ ssd1-d2 RAD5 leo1::KANMX*	This study
8737	*MATα ade2-1 trp1-1 can1-100 leu2-3,112 his3-11,15 ura3 GAL+ psi+ ssd1-d2 RAD5 leo1::KANMX*	This study
8743	*MATa ade2-1 trp1-1 can1-100 leu2-3,112 his3-11,15 ura3 GAL+ psi+ ssd1-d2 RAD5 rtf1::KANMX*	This study
8744	*MATalpha ade2-1 trp1-1 can1-100 leu2-3,112 his3-11,15 ura3 GAL+ psi+ ssd1-d2 RAD5 rtf1::KANMX*	This study
8751	*MATa ade2-1 trp1-1 can1-100 leu2-3,112 his3-11,15 ura3 GAL+ psi+ ssd1-d2 RAD5 ctr9::KANMX*	This study
8752	*MATalpha ade2-1 trp1-1 can1-100 leu2-3,112 his3-11,15 ura3 GAL+ psi+ ssd1-d2 RAD5 ctr9::KANMX*	This study
8757	*MATa ade2-1 trp1-1 can1-100 leu2-3,112 his3-11,15 ura3 GAL+ psi+ ssd1-d2 RAD5 paf1::KANMX*	This study
8758	*MATalpha ade2-1 trp1-1 can1-100 leu2-3,112 his3-11,15 ura3 GAL+ psi+ ssd1-d2 RAD5 paf1::KANMX*	This study
8803	*MATa ade2-1 trp1-1 can1-100 leu2-3,112 his3-11,15 ura3 GAL+ psi+ ssd1-d2 RAD5 vps36::KANMX*	This study
8804	*MATα ade2-1 trp1-1 can1-100 leu2-3,112 his3-11,15 ura3 GAL+ psi+ ssd1-d2 RAD5 vps36::KANMX*	This study
9191	*MATa ade2-1 trp1-1 can1-100 leu2-3,112 his3-11,15 ura3 GAL+ psi+ ssd1-d2 RAD5 cdc73(41-90)::KANMX*	This study
9192	*MATα ade2-1 trp1-1 can1-100 leu2-3,112 his3-11,15 ura3 GAL+ psi+ ssd1-d2 RAD5 cdc73(41-90)::KANMX*	This study
9195	*MATa ade2-1 trp1-1 can1-100 leu2-3,112 his3-11,15 ura3 GAL+ psi+ ssd1-d2 RAD5 vps36(1302-1701)-cdc73(782-1182)::KANMX*	This study
9196	*MATalpha ade2-1 trp1-1 can1-100 leu2-3,112 his3-11,15 ura3 GAL+ psi+ ssd1-d2 RAD5 vps36(1302-1701)-cdc73(782-1182)::KANMX*	This study
9200	*MATalpha ade2-1 trp1-1 can1-100 leu2-3,112 his3-11,15 ura3 GAL+ psi+ ssd1-d2 RAD5 vps36(41-90)::KANMX*	This study
9201	*MATa ade2-1 trp1-1 can1-100 leu2-3,112 his3-11,15 ura3 GAL+ psi+ ssd1-d2 RAD5 vps36(1-90)::KANMX cdc13-1*	This study
11766	*MATalpha vps27::HPH ade2-1 trp1-1 can1-100 leu2-3,112 his3-11,15 ura3 GAL+ psi+ ssd1-d2 RAD5*	This study
11767	*Mata vps27::HPH ade2-1 trp1-1 can1-100 leu2-3,112 his3-11,15 ura3 GAL+ psi+ ssd1-d2 RAD5*	This study
11773	*MATa stp22::HPH ade2-1 trp1-1 can1-100 leu2-3,112 his3-11,15 ura3 GAL+ psi+ ssd1-d2 RAD5*	This study
11774	*MATalpha stp22::HPH ade2-1 trp1-1 can1-100 leu2-3,112 his3-11,15 ura3 GAL+ psi+ ssd1-d2 RAD5*	This study
10414	*MATa ade2-1 trp1-1 can1-100 leu2-3,112 his3-11,15 ura3 GAL+ psi+ ssd1-d2 RAD5 snf8::HYG*	This study
10415	*MATalpha ade2-1 trp1-1 can1-100 leu2-3,112 his3-11,15 ura3 GAL+ psi+ ssd1-d2 RAD5*	This study

	snf8::HYG	
10422	*MATa ade2-1 trp1-1 can1-100 leu2-3,112 his3-11,15 ura3 GAL+ psi+ ssd1-d2 RAD5 vps25::NAT*	This study
10423	*MATalpha ade2-1 trp1-1 can1-100 leu2-3,112 his3-11,15 ura3 GAL+ psi+ ssd1-d2 RAD5 vps25::NAT*	This study
11783	*MATalpha snf7::HPH ade2-1 trp1-1 can1-100 leu2-3,112 his3-11,15 ura3 GAL+ psi+ ssd1-d2 RAD5*	This study
11784	*Mata snf7::HPH ade2-1 trp1-1 can1-100 leu2-3,112 his3-11,15 ura3 GAL+ psi+ ssd1-d2 RAD5*	This study

Table S2- List of the primers used in this study.

**Primer**	**Annealing region**	**Sequence**	**Used for**	**Ref.**
3275	*BUD6*	GACCGGGCACATTTAATCAG	RT-qPCR internal control	(Rodrigues and Lydall 2018)
3276	*BUD6*	TCAGCCTTGTCAATAGCTTCG	RT-qPCR internal control	(Rodrigues and Lydall 2018)
3261	*TLC1*	AATGTGCCCCGTACATCGAA	RT-qPCR	This study.
3262	*TLC1*	CGCAAACCTAACCGATGCTT	RT-qPCR	This study.
3271	*VPS36*	GAACTCGCTTGGGTTGGAAT	RT-qPCR	This study.
3272	*VPS36*	GGGGTCCTTGAAAAACAGGA	RT-qPCR	This study.
4700	*VPS27*	TGCAAAATCCAGCAGAAAGC	RT-qPCR	This study.
4701	*VPS27*	TTACCGATTGTTGGCGTGAG	RT-qPCR	This study.
4702	STP22	TAGACGTCCTGACCGCAAAT	RT-qPCR	This study.
4703	STP22	TCTGTTTTGGCCACTGCTATG	RT-qPCR	This study.
4708	SNF7	AGGGAGCAAGTCGAATTAGGA	RT-qPCR	This study.
4709	SNF7	ATTCCTCGTCCAGCTCATCTT	RT-qPCR	This study.

Rodrigues J, Lydall D. 2018. Paf1 and Ctr9, core components of the PAF1 complex, maintain low levels of telomeric repeat containing RNA. *Nucleic acids research* **46**: 621-634.
